# Betaine and nano-emulsified vegetable oil supplementation for improving carcass and meat quality characteristics of broiler chickens under heat stress conditions

**DOI:** 10.3389/fvets.2023.1147020

**Published:** 2023-03-27

**Authors:** Gamaleldin M. Suliman, Elsayed O. S. Hussein, Abdullah N. Al-Owaimer, Rashed A. Alhotan, Maged A. Al-Garadi, Jameel M. H. Mahdi, Hani A. Ba-Awadh, Mohammed M. Qaid, Ayman Abdel-Aziz Swelum

**Affiliations:** ^1^Department of Animal Production, College of Food and Agriculture Sciences, King Saud University, Riyadh, Saudi Arabia; ^2^Department of Zoology, College of Science, King Saud University, Riyadh, Saudi Arabia

**Keywords:** betaine, magic oil, carcass traits, meat quality, heat stress, broiler chickens

## Abstract

**Introduction:**

This research aimed to examine the effects of water-added betaine (BET) and/or nano-emulsified vegetable oil (MAGO) on carcass and meat quality characteristics of broilers raised under thermoneutral (TN) and heat stress (HS) conditions.

**Methods:**

On day 21, 640 birds (Ross 308) were randomly assigned to one of two thermal conditions (thermoneutral 22 ± 1°C and heat stress 32 ± 1°C) each containing four treatment groups: Control, BET, MAGO, and a mixture of both (BETMAGO) in a 2 × 4 factorial arrangement (eight groups). Each group has eight replicates, with ten birds each. The birds' carcass and meat quality characteristics were evaluated at 35 days.

**Results and discussion:**

The dressing percentage, breast, leg, wing, heart, initial pH, color change, cooking loss (CL), water-holding capacity (WHC), shear force (SF), and texture profile with exception of springiness significantly affected by the treatments. The results showed that HS had negative effects on carcass weight and relative weights of the breast, spleen, and heart. Moreover, HS increased dressing percentage, wing, initial pH, final core temperature, initial lightness, WHC, and hardness. Significant differences in interactions between treatments and temperature were observed in the spleen, WHC, and SF.

**Conclusion:**

Water supplemented with BET effectively improved carcass dressing percentage, breast weight, and meat quality in terms of water-holding capacity and tenderness under HS conditions. More studies on the use of BET and/or MAGO at different levels were recommended.

## Introduction

Poultry meat is generally considered a rich source of nutrients, including high-quality proteins, vitamins, and minerals. Additionally, it has a low unsaturated fatty acid profile, making it an optimal dietary choice for all age groups (1). Poultry provides humans with nutrition and fiber through meat, eggs, and feathers.

The poultry industry has expanded quickly in Saudi Arabia during the past few decades. It has the potential to attract a considerable number of investments within a short period of time, garner government support to achieve self-sufficiency, compete with imported products, and address the considerable increase in the rate of consumption of poultry meat in the Kingdom of Saudi Arabia over the past few years. However, heat stress is a major concern in the poultry industry, affecting broiler chickens' mass gain and immunity (2). In addition, chronic heat stress has a negative impact on the quality of poultry meat (3). Supplementing Magic oil and probiotics is recommended for the optimal growth of broilers in poultry programs, particularly for male birds, during the period of 0 to 30 days of age (4). It has been found that supplementing male broilers with Magic oil and probiotics, especially during the first 30 days of their lives, can result in good meat chewiness due to higher springiness and lower hardness and cohesiveness. Furthermore, these supplements were found to be associated with the most convenient cooking loss value. Natural herbs or their derivatives, such as *Rumex nervosus* leaves, used as phytogenic feed additives improved meat quality and some carcass traits in broilers and are believed to be a promising non-traditional feed source in the future (5).

Betaine, or tri-methyl glycine, is a naturally occurring chemical compound that serves as an organic osmolyte in microbes and flora exposed to osmotic stress and dehydration (6). Under severe osmotic stress, water migrates from the cell toward higher concentrations of solutes outside the cell (7–10). The constant loss of water causes cells to shrink and eventually die. As a result, osmolytes must regulate osmotic pressure to conserve water and maintain cell integrity.

Several studies have shown that natural antioxidants supplied before and after slaughter improve carcass traits, shelf life, and meat quality. For example, natural antioxidants can be added to animal feed or drinking water, applied to the meat surface, or used in active packaging (11). However, supplementation of these additives in feed or drinking water has been proven to be a better method than other strategies (12, 13), as it allows animals to spread the substance more effectively in the body and absorb tolerable and safe levels of the compound (14). Nanoemulsions are used in food manufacturing to encapsulate, protect, deliver, and transport hydrophobic (poorly water-soluble) bioactive components such as nutrients, nutraceuticals, antimicrobials, and antioxidants (15, 16). They consist of tiny oil droplets suspended in water that serve as vehicles for essential oil bioavailability (17). The pH of meat is an important factor in determining meat quality because it influences the color, texture, and WHC of the breast meat when the muscles are stiff. Within the first 15 m following death, pH plays a significant role in determining the shelf life of meat products (18).

Abudabos et al. (19) and Ghanima et al. (20) have investigated the use of nano-emulsified vegetable oil as a Magic oil in the diets of broiler chickens and rabbits (20). However, few studies have explored the potential benefits of supplementing drinking water with this oil. As a result, there is a need to investigate the effects of adding these supplements to drinking water and assess their effectiveness. Thus, this experiment aimed to investigate the effects of water-added betaine and/or Magic oil on the carcass traits and meat quality of broiler chickens subjected to thermal stress conditions.

## Materials and methods

### Ethical approval

The experiment was conducted in accordance with the code of ethics and guidelines for the use of animals in research, and the protocol was approved by the King Saud University Ethics Committee (Approval No. KSU-SE-21-02).

### Chemical specifications of Magic oil™ and Betafin^®^ S6

Magic oil™ (Atcopharma El-Menofia, Egypt) contains 98.5% nano-emulsified crude oil, including 26% monounsaturated fat, 59% polyunsaturated fat (50% linoleic acid; n-6 & 7% linolenic acid; n-3), 14% saturated fat, and vitamin E.

Betafin^®^ S6 (Danisco Animal Nutrition, Marlborough, Wiltshire SN81XN, United Kingdom) is a natural betaine ideal for drinking water applications. It contains ≥ 91% anhydrous betaine [1-Carboxy-N, N, N-trimethylolethane-aminium hydroxide inner salt (trimethylglycine)] with ≥ 5% anti-caking agent (calcium stearate), moisture (halogen drying) ≤ 2 %, chloride (IC) ≤ 1,000 ppm, sulfate (IC) ≤ 1,000 ppm, and heavy metals ≤ 10 ppm.

### Bird's management and treatment groups

The experiment was conducted in the Animal Production Department, College of Food and Agricultural Sciences, KSU, Saudi Arabia. The broiler chicks (Ross 308) were procured from the Alkhumasia commercial hatchery in < city>Riyadh < /city>, Saudi Arabia, and individually weighed to ensure they were assigned to similar groups (46.3 g ± 0.13). The broiler chicks were raised under the recommended temperatures, with the initial brooding temperature set at 33°C on day 0. This was gradually decreased by 1°C per week until it reached 24°C on day 20. On day 21, a total of 640 straight-run broiler chicks with comparable body weights (1,010 g ± 11.56) were selected and randomly assigned to 64-floor cage pens (100 cm width, 100 cm length) located in two environmentally controlled rooms. Each cage with 10 birds was considered the experimental unit. Therefore, a 2 × 4 factorial arrangement was applied. Eight experimental groups (eight replicates per group) were formed by using two conditions with rising temperatures [thermoneutral (TN) and constant heat stress (HS)] and four water-added supplementation treatments (control, BET, MAGO, and BETMAGO). The birds in one of the two experimental rooms were continuously kept under the TN condition (22 ± 1°C), while the birds in another room were raised under constant heat stress conditions (HS; 32 ± 1°C). The birds were given one of four water additive treatments: control (no additives), Magic oil (MAGO) at a rate of 1 mL/L drinking water, betaine (BET) at a rate of 1 g/L drinking water, or a mixture of Magic oil and betaine (MAGOBET: a mixture of 1 g betaine and 1 mL Magic oil/L drinking water). The eight treatment groups were as follows: group 1, TN + CON; group 2, TN + 1 g/L drinking water MAGO; group 3, TN + 1 g/L drinking water BET; group 4, TN + 1 g/L drinking water MAGOBET combination; group 5, HS + CON; group 6, HS + 1 g/L drinking water MAGO; group 7, HS + 1 g/L drinking water BET; and group 8, HS + 1 g/L drinking water MAGOBET combination. The presence of HS has a negative impact on the slaughter weight, carcass weight, internal organ weights, and quality of poultry meat (3). Therefore, in the current study, half of the broilers were exposed to the HS challenge during the finisher period, while the other half were not (20–35 days). Therefore, during the same period of the HS challenge, from 20 to 35 days, all birds under TN and HS conditions received water supplements with Magic oil and betaine Magic oil and betaine combination to mitigate the adverse effects of chronic heat stress in stressed broilers and compare their effects with those of the birds reared under TN conditions. The lighting protocol was set at 22 h of light and 2 h of darkness per day.

To meet the nutritional recommendations for the Ross 305 strain, standard experimental diets were formulated based on commercial practice recommendations in Saudi Arabia (Table 1). These diets were divided into three feeding periods: starter (0–10 d), grower (11–20 d), and finisher (21–35 d), which corresponded to changes in the birds' protein and energy requirements as they aged. The diets were given in pellet form and were made using a corn-soybean meal (CSBM) base.

**Table 1:** Composition of experimental diets and nutrients analysis.

**Ingredients (%)**	**Starter (0–10 d)**	**Grower (11–20 d)**	**Finisher (21–35 d)**
Corn grain	57.65	61.32	60.64
Soybean meal (48% CP)	35.43	31.79	30.48
Palm oil	2.06	2.61	4.63
Dical phos	1.92	1.73	1.42
Limestone	1.06	0.96	1.42
Common salt	0.36	0.36	0.35
L-Threonine	0.30	0.12	0.07
Methionine	0.38	0.33	0.28
LLysine HCl	0.28	0.22	0.15
Choline Cl70	0.06	0.06	0.06
Minvit Arasco 0.5%	0.50	0.50	0.50
**Nutrients**		**Analysis**	
Dry Matter %	89.614	89.576	85.664
ME kcal/kg	3000	3070	3200
CP %	22.4	20.7	19.5
Arginine %	1.52	1.399	1.273
Lysine %	1.435	1.29	1.131
Methionine %	0.711	0.641	0.566
Methionine + Cysteine %	1.08	0.99	0.882
Threonine %	1.143	0.9	0.803
Tryptophan %	0.277	0.254	0.228
Valine %	1.017	0.951	0.901
Ether extract	4.601	5.256	7.236
Linoleic acid %	1.597	1.714	4.496
Crude Fiber %	2.65	2.589	2.523
Calcium %	0.96	0.87	0.937
Total phosphorus %	0.713	0.663	0.625
Available phosphorus %	0.48	0.44	0.4
Sodium %	0.16	0.16	0.16

### Carcass measurements

On day 35, 16 birds were chosen at random from each group. Feathers, heads, and shanks were removed after slaughter, and the residual carcasses were sectioned to split the breast and thigh. Fat, liver, heart, gizzard, wings, and drumstick were all separated and weighed in the same way. The percentage of yield for each part was calculated using dressing weight (21).

### Meat characteristics

#### PH and temperature

pH and temperature were measured directly in the breast sample after 1 h postmortem using a microprocessor *pH*-meter (Model PH 211, Hanna Instruments). Two readings were conducted for each carcass, and the mean value was computed.

#### Meat color

The CIELAB Color System is a color space used to describe color. L^*^ represents lightness, a^*^ represents the degree of redness, and b^*^ represents the degree of yellowness. Color measurements were taken on the breast meat 15 min after slaughter using a chroma meter (Konica Minolta, CR-400-Japan). Additional color parameters were calculated, including color change (ΔE), b/a ratio, chroma (C), and hue angle (A°) (22).

#### Cooking loss

The frozen breast muscle (~100 g) was thawed overnight at 4°C. Once the internal temperature reached 70°C, the meat was cooked on a commercial tabletop grill. A thermocouple thermometer (Ecoscan Temp JKT, Eutech Instruments) was inserted into the center of the muscle to measure the cooking temperature. Cooking loss was calculated as the difference between the initial and final weight of the muscles by weighing them before and after cooking.

#### Shear force

The shear force was measured using a cooked meat sample that had been subjected to an earlier handling process to determine the amount of cooking loss, as outlined by Wheeler et al. (23). Five round cores measuring 1.27 cm in diameter were then removed from each muscle sample that was parallel to the direction of the muscle fibers after they had been cooled to room temperature (21°C). With the aid of a handheld coring tool, cores were obtained. Using a texture analyzer with a Warner-Bratzler attachment, the Texture Analyzer (TA-HD-Stable MicroSystems, England) was used to calculate the SF, which was defined as the maximum force (N) vertical to the fibers. The crosshead speed that was chosen was 200 mm/min.

#### Water-holding capacity (WHC)

The Wilhelm et al. (24) method was used to calculate WHC. For each sample, the pectoral muscle was divided into two replicates weighing ~2 g each. The samples were then sandwiched between two Plexiglas disks and two filter papers and placed under a 10 kg weight for 5 m. The sample was then weighed, and the difference between the initial and final weights was used to calculate the WHC.

### Statistical analysis

All analysis was carried out using the Statistical Analysis System (25) in a two-way ANOVA. The experimental design was a randomized complete block design (RCBD) with room “raising conditions” (*n* = 2) as a blocking factor. Means for variables showing significant differences were tested using the PDIFF option. The probability value was set at *P* < 0.05. Means ± standard errors of the mean (SEM) were used to express all values.

## Results

### Carcass traits

The HS effects and experimental treatments and their interactions on carcass characteristics and the relative weights of body components of broiler chickens at day 35 are presented in Table 2. Treatments, HS, or their interaction had no effect (*P* > 0.05) on the relative weights of carcass characteristics and the relative weights of the body components of the broiler chickens.

**Table 2:** Effect of the treatments on carcass characteristics and body components of broiler chickens at d 35 under thermoneutral (TN) and heat stress (HS) raising conditions.

**Treatments (TRT)**	**Raising Condition (RC)**	**Final LW (kg)**	**HCW (kg)**	**CCW (kg)**	**Dressing (%)**	^**1**^ **Body components were computed as a ratio to the carcass weight**							
						**Breast %**	**Leg %**	**Wing %**	**Gizzard (%)**	**Liver %**	**Spleen %**	**Heart %**	**Abdominal fat %**
Effect of interactions between treatments and raising condition (TRT*RC)													
Control^1^	TN	2.43	1.83	1.85	75.23	48.96	37.60	9.03	1.83	2.68	0.14	0.67	1.73
MAGO^2^	TN	2.38	1.80	1.79	75.62	47.57	37.89	9.37	1.73	2.65	0.18	0.63	1.61
BET^3^	TN	2.53	1.95	1.93	76.99	49.50	36.51	8.65	1.73	2.67	0.17	0.62	1.70
BETMAGO^4^	TN	2.43	1.85	1.85	76.19	48.70	38.44	9.10	1.81	2.57	0.14	0.65	1.68
Control	HS	2.05	1.57	1.56	76.77	47.54	37.45	9.41	1.89	3.31	0.12	0.61	1.74
MAGO	HS	2.06	1.59	1.58	77.30	46.59	38.53	9.66	1.90	2.39	0.10	0.53	1.68
BET	HS	2.11	1.65	1.64	78.08	48.38	37.01	9.00	1.71	2.25	0.10	0.53	1.82
MAGOBET	HS	2.16	1.67	1.66	77.41	46.93	37.78	9.45	1.73	2.13	0.11	0.52	1.64
SEM		62.60	50.63	48.54	0.45	0.632	0.485	0.148	0.06	0.30	0.01	0.03	0.12
*p-values*		0.668	0.678	0.600	0.912	0.929	0.514	0.983	0.196	0.184	0.002	0.523	0.946
**Effect of treatments (TRT)**													
Control		2.24	1.70	1.71	76.00^b^	48.25^ab^	37.52^ab^	9.22^ab^	1.86	3.00	0.13	0.64^a^	1.73
MAGO		2.22	1.70	1.68	76.46^b^	47.08^b^	38.21^a^	9.51^a^	1.82	2.52	0.14	0.58^b^	1.65
BET		2.32	1.80	1.78	77.54^a^	48.94^a^	36.76^b^	8.82^b^	1.72	2.46	0.13	0.58^b^	1.76
BETMAGO		2.30	1.76	1.75	76.80^ab^	47.82^ab^	38.11^a^	9.27^a^	1.77	2.35	0.12	0.59^b^	1.66
SEM		44.26	35.80	34.32	0.32	0.45	0.45	0.34	0.11	0.04	0.21	0.01	0.02
*p-values*		0.346	0.141	0.153	0.008	0.032	0.011	< 0.0001	0.130	0.131	0.415	0.043	0.735
**Effect of raising condition (RC)**													
TN^5^		2.444^a^	1.858^a^	1.854^a^	76.01^b^	48.68^a^	37.61	9.03^b^	1.78	2.65	0.16^a^	0.64^a^	1.68
HS^6^		2.093^b^	1.621^b^	1.607^b^	77.39^a^	47.36^b^	37.69	9.38^a^	1.81	2.52	0.11^b^	0.55^b^	1.72
SEM		31.30	25.32	24.27	0.23	0.32	0.24	0.07	0.03	0.15	0.01	0.01	0.06
*p-values*		< 0.0001	< 0.0001	< 0.0001	< 0.0001	0.004	0.790	0.001	0.504	0.542	< 0.0001	< 0.0001	0.772

FLW, Final live weight; HCW, Hot carcass weight; CCW, Cold carcass weight; ^a−*c*^Mean values of different superscripts on the same column are significantly different at (P < 0.05). ^1^Control, no additive; ^2^MAGO, Magic oil (1 mL/L drinking water); ^3^BET, betaine (1 g/L drinking water); ^4^BETMAGO, a mixture of the 1 g betaine and 1 mL Magic oil/L drinking water; ^5^TN, thermoneutral environment (22°C); ^6^HS, heat stress environment 33°C, SEM, Standard Error of Mean.

The treatments had significant effects on the dressing percentage (*P* = 0.008) relative to the weights of the breast (*P* = 0.032), leg (*P* = 0.011), wing, and heart (*P* < 0.0001). BET supplementation increased the percentage of hot carcass dressing compared to the control. Compared to the control, the treatments reduced the relative weight of the heart. Except for the spleen, the treatment^*^temperature interaction did not affect the body components' relative weight.

HS reduced the broiler chickens' final live weight, hot and cold carcass weight, and relative weights of the breast, spleen, and heart (*P* < 0.05). In contrast, HS increased the percentage of hot carcass dressing and the relative weight of the broiler chickens' wings (*P* < 0.05).

### Meat quality

The initial and final pH and temperature of the breast samples from 35-day-old broiler chickens are shown in Table 3. Treatments and rising temperatures had significant interactional effects on the initial pH of breast meat (*P* < 0.05). The treatments and rising temperatures had no impact on the ultimate pH (*P* > 0.05). However, the ultimate pH significantly differed due to their interaction (*P* = 0.001). Stressed birds had a higher initial pH (6.24 *vs*. 6.10, *P* < 0.0001), lower initial core temperature (21.69 *vs*. 29.20°C, *P* < 0.0001), and a higher final core temperature (17.95 *vs*. 16.96°C, *P* < 0.0001) of the breast muscle compared to the unstressed birds.

**Table 3:** Effect of the treatments on the pH and core temperature at 1 and 24 h postmortem of breast muscle of broiler chickens at d 35 under thermoneutral (TN) and heat stress (HS) raising conditions.

**Treatments (TRT)**	**1 h P.M**			**24 h P.M**	
	**Raising condition (RC)**	**pH**	**Temp** °**C**	**pH**	**Temp** °**C**
Effect of interactions between treatments and raising condition (TRT*RC)					
Control^1^	TN	6.07	30.01	5.89	16.90
MAGO^2^	TN	6.16	29.55	5.78	17.26
BET^3^	TN	6.06	28.80	5.80	16.84
BETMAGO^4^	TN	6.13	28.43	5.88	16.86
Control	HS	6.26	22.36	5.85	17.78
MAGO	HS	6.34	22.00	5.87	18.21
BET	HS	6.23	20.76	5.88	17.91
MAGOBET	HS	6.13	21.64	5.83	17.90
SEM		0.03	0.72	0.02	0.17
*p-values*		0.011	0.851	0.001	0.934
**Effect of treatments (TRT)**					
Control		6.17^b^	26.18	5.87	17.34
MAGO		6.26^a^	25.78	5.82	17.73
BET		6.14^b^	24.78	5.84	17.37
BETMAGO		6.13^b^	25.03	5.85	17.38
SEM		0.02	0.51	0.02	0.12
*p-values*		0.001	0.184	0.171	0.060
**Effect of raising condition (RC)**					
TN^5^		6.10^b^	29.20^a^	5.84	16.96^b^
HS^6^		6.24^a^	21.69^b^	5.86	17.95^a^
SEM		0.02	0.36	0.01	0.08
*p-values*		< 0.0001	< 0.0001	0.199	< 0.0001

^a−*c*^Mean values of different superscripts on the same column are significantly different at (P < 0.05). ^1^Control, no additive; ^2^MAGO, Magic oil (1 mL/L drinking water); ^3^BET, betaine (1 g/L drinking water); ^4^BETMAGO, a mixture of the 1 g betaine and 1 mL Magic oil/L drinking water; ^5^TN, thermoneutral environment (22°C); ^6^HS, heat stress environment 33°C, SEM, Standard Error of Mean.

The initial and final meat color derivatives of breast samples from 35-day-old broiler chickens are shown in Table 4. Although the color change was noticeably amplified in the birds that were given BET or MAGO alone, water supplementation treatments had no significant main or additive effects on breast color. The L^*^ value increased over time (1 and 24 h postmortem) in the breasts of the chickens that were given experimental water treatment.

**Table 4:** Effect of the treatments on meat color derivatives of breast muscle of broiler chickens at d 35 under thermoneutral (TN) and heat stress (HS) raising conditions.

**Treatments (TRT)**	**Raising condition (RC)**	Li*1	ai*1	bi*1	Lu*2	au*2	bu*2	**Color change**	**b:a ratio**	**Chroma**	**Hue angle**
Effect of interactions between treatments and raising condition (TRT*RC)											
Control^1^	TN	49.72	−1.08	8.58	53.33	−1.06	11.01	5.05	−4.62	11.12	−50.45
MAGO^2^	TN	51.24	−1.29	8.12	54.70	−1.00	11.72	6.34	−8.23	11.84	−62.54
BET^3^	TN	50.74	−1.18	8.47	53.62	−1.14	11.59	5.74	−3.36	11.71	−50.19
BETMAGO^4^	TN	50.37	−1.39	9.08	53.30	−0.77	11.54	4.66	−5.56	11.62	−41.07
Control	HS	52.04	−0.94	8.15	53.05	−0.47	10.99	4.13	−6.05	11.07	−43.03
MAGO	HS	50.70	−1.34	8.67	50.16	−0.38	11.18	4.73	−8.27	11.37	−43.13
BET	HS	52.49	−1.33	7.56	50.37	−0.63	10.06	4.88	−7.19	10.16	−52.70
MAGOBET	HS	51.40	−1.54	8.07	50.64	−1.10	10.27	3.62	−6.58	10.38	−61.46
SEM		0.56	0.22	0.36	0.94	0.34	0.47	0.53	3.96	0.46	16.86
*p-values*		0.07	0.90	0.13	0.15	0.45	0.36	0.89	0.97	0.34	0.68
**Effect of treatments (TRT)**											
Control		50.88	−1.01	8.37	53.19	−0.77	11.00	4.59^ab^	−5.33	11.10	−46.74
MAGO		50.97	−1.31	8.39	52.43	−0.69	11.45	5.53^a^	−8.25	11.61	−52.84
BET		51.61	−1.26	8.02	51.99	−0.88	10.83	5.31^a^	−5.28	10.94	−51.45
BETMAGO		50.88	−1.47	8.57	51.97	−0.93	10.90	4.14^b^	−6.07	11.00	−51.26
SEM		0.40	0.16	0.25	0.67	0.24	0.33	0.38	2.80	0.33	11.92
*p-values*		0.50	0.23	0.47	0.53	0.89	0.55	0.04	0.86	0.46	0.99
**Effect of raising condition (RC)**											
TN^5^		50.52^b^	−1.23	8.56^a^	53.74^a^	−0.99	11.47^a^	5.45^a^	−5.44	11.58^a^	−51.06
HS^6^		51.66^a^	−1.29	8.11^b^	51.05^b^	−0.65	10.62^b^	4.34^b^	−7.02	10.75^b^	−50.08
SEM		0.28	0.11	0.18	0.47	0.17	0.23	0.27	1.98	0.23	8.43
*p-values*		0.01	0.74	0.08	< 0.0001	0.16	0.01	0.01	0.57	0.01	0.93

^a−*c*^Mean values of different superscripts on the same column are significantly different at (P < 0.05). ^1^Control, no additive; ^2^MAGO, Magic oil (1 mL/L drinking water); ^3^BET, betaine (1 g/L drinking water); ^4^BETMAGO, a mixture of the 1 g betaine and 1 mL Magic oil/L drinking water; ^5^TN, thermoneutral environment (22°C); ^6^HS, heat stress environment 33°C, SEM, Standard Error of Mean.

When compared to the unstressed birds, the heat-stressed birds had higher initial lightness (51.66 *vs*. 50.52, *P* = 0.005) and lower final lightness (53.74 *vs*. 51.05, *P* < 0.0001). Furthermore, the heat-stressed birds had lower initial yellowness (8.11 *vs*. 8.56, *P* = 0.081) and final yellowness (10.62 *vs*. 11.47, *P* = 0.012) of the breast muscle than the unstressed birds. Color change (4.34 *vs*. 5.45, *P* = 0.004) and chroma saturation (10.75 *vs*. 11.58, *P* = 0.012) were lower in the HS-exposed birds than in the HS-unexposed birds.

The WHC, CL, SF, and texture profile analysis (TPA) of breast samples from 35-day-old broiler chickens are presented in Table 5. The WHC, CL, SF, and texture profile analysis (TPA) of breast samples are significantly (P < 0.05) influenced by treatments and/or rising temperatures. However, the values for springiness were unaffected (*P* > 0.05) by the water treatments. Furthermore, the thermal condition had no effect on CL or chewiness (*P* > 0.05). The interactions between water treatments and thermal conditions had no effect (*P* > 0.05) on all meat parameters, except for WHC and SF.

**Table 5:** Effect of the treatments on chicken breast objective eating quality measures at d 35 under thermoneutral (TN) and heat stress (HS) raising conditions.

**Treatments (TRT)**	**Raising condition (RC)**	**WHC (%)**	**CL (%)**	**SF (N)**	**Texture profile analysis (TPA)**			
					**Hardness (N)**	**Springiness**	**Cohesiveness**	**Chewiness**
**Effect of interactions between treatments and raising condition (TRT** ^*^ **RC)**								
Control^1^	TN	29.97	16.28	7.18	7.43	0.85	0.51	3.42
MAGO^2^	TN	32.58	21.95	6.50	7.34	0.83	0.51	3.30
BET^3^	TN	33.94	19.47	5.32	7.80	0.86	0.55	3.79
BETMAGO^4^	TN	31.15	21.55	9.26	7.91	0.83	0.55	3.68
Control	HS	35.17	16.28	4.81	7.75	0.77	0.47	2.81
MAGO	HS	31.20	24.15	5.07	9.23	0.75	0.51	3.79
BET	HS	36.31	19.47	4.94	10.07	0.75	0.52	4.13
MAGOBET	HS	31.83	21.55	5.15	9.45	0.76	0.52	3.89
SEM		0.77	1.82	0.36	0.45	0.02	0.02	0.27
*p-values*		< 0.0001	0.91	< 0.0001	0.16	0.72	0.72	0.14
**Effect of treatments (TRT)**								
Control		32.57^b^	16.28^b^	5.99^b^	7.59^b^	0.81	0.49^b^	3.12^b^
MAGO		31.89^b^	23.05^a^	5.79^bc^	8.28^ab^	0.79	0.51^ab^	3.55^ab^
BET		35.13^a^	19.47^ab^	5.13^c^	8.93^a^	0.80	0.54^a^	3.96^a^
BETMAGO		31.49^b^	21.55^a^	7.21^a^	8.68^a^	0.79	0.54^a^	3.78^a^
SEM		0.54	1.28	0.26	0.32	0.01	0.01	0.18
*p-values*		< 0.0001	0.002	< 0.0001	0.02	0.70	0.04	0.01
**Effect of raising condition (RC)**								
TN^5^		31.91^b^	19.81	7.07^a^	7.62^b^	0.84^a^	0.53^a^	3.55
HS^6^		33.62^a^	20.36	4.99^b^	9.13^a^	0.76^b^	0.51^b^	3.65
SEM		0.39	0.91	0.18	0.23	0.01	0.01	0.13
*p-values*		0.002	0.67	< 0.0001	< 0.0001	< 0.0001	0.05	0.56

^a−*c*^Mean values of different superscripts on the same column are significantly different at (P < 0.05). WHC, water-holding capacity; CL, cooking loss; ^1^Control, no additive; ^2^MAGO, Magic oil (1 mL/L drinking water); ^3^BET, betaine (1 g/L drinking water); ^4^BETMAGO, a mixture of the 1 g betaine and 1 mL Magic oil/L drinking water; ^5^TN, thermoneutral environment (22°C); ^6^HS, heat stress environment 33°C, SEM, Standard Error of Mean.

WHC values of breast meat were higher in the HS-exposed chickens (33.62 *vs*. 31.91%, *P* = 0.002) and those who were given BET (36.31). Nevertheless, there was evidence of an interaction between the temperature regime and BET, as seen in the breasts of the birds that were fed BET (36.31 *vs*. 33.94%) rather than those who were in the control group (35.17 *vs*. 29.97%). Cooking loss was significantly (*P* = 0.002) higher in experimentally treated birds' breast meat than in the control group.

The lesser shear force value was reported by the BET treatment group (5.13 N), resulting in the lowest shear force value of chickens that drank BET and were housed under HS conditions (4.94 N), indicating a significant level of tenderness., SF and shear energy are the maximum force and energy needed to cut the sample; thus, lower values indicate that the meat was more tender. The highest value of shear force was higher in the TN-exposed chickens (7.07 *vs*. 4.99 N, *P* < 0.0001) and was higher in the birds that received BETMAGO treatment (7.21 N, *P* < 0.0001), indicating tough meat, resulting in treatment^*^thermal interaction, as shown by the BETMAGO treatment group under TN (9.26 N). In the texture profile analysis, the BET and then BETMAGO treatments had the highest values for hardness, cohesiveness, and chewiness.

## Discussion

This study compared the efficacy of Magic oil, a natural nano-emulsified vegetable oil, with betaine on carcass characteristics and breast quality in broiler chickens. In this case, BET increased the dressing percentage and breast percentage significantly. This suggests that supplementing BET with water improved breast weight, which increased the dressing percentage by alleviating the negative effects of HS. The addition of 0.05% methionine increased meat yield by ~1.5 percentage points, whereas 0.04% betaine only increased breast meat yield by 0.3 to 0.6 percentage points (26). They concluded that there was no evidence to suggest that betaine served as a replacement for DL-methionine as an essential amino acid supplement in the broiler chickens' diets. However, BET supplementation improved carcass values slightly. Additionally, the study found that BET supplementation improved the yield of breast muscle compared to the control group. The findings of Waldroup et al. (27) and McDevitt et al. (28) are also consistent with our findings. BET supplementation improved carcass dressing by increasing slaughter and raising carcass weights more than BETMAG, indicating that BET alone is a preferable supplement without the need for MAG. Waldroup and Fritts (29) found a rise in dressing percentage in the broilers that were fed 0.1% BET at 42 days. Furthermore, when compared to TN, HS reduced breast yield. Temperature also had an effect on the relative weight of the spleen, which was significantly lower in the broilers raised under HS than in those raised under TN. According to recent studies, lymphoid organ growth in heat-stressed broilers has slowed (19, 30, 31). In addition, the weight loss could be due to a decrease in feed intake, which results in fewer nutrients for the appropriate growth of lymphoid organs under HS conditions (32).

Meat pH is an important parameter in determining meat quality that occurs during muscle stiffness; it can affect the texture, color, and WHC of meat. pH is an important factor in determining the shelf life of meat products within the first 15 m after death (18). Usually, a rapid drop in meat pH can cause protein denaturation, resulting in pale meat with low WHC. Several studies have suggested that nano-emulsion-based products can positively influence the physicochemical and sensory properties of the breast muscles (33). To the best of our knowledge, to date, there have not been many studies that examine how nanoemulsified vegetable oil affects meat characteristics. In a current study, BET seemed to have a greater impact on the physicochemical properties of filets than MAGO. According to Smith et al. (34), breast meat color was unaffected by BET, but HS increased L^*^ and decreased b^*^. According to Akşit et al. (35), the broilers raised under cyclic HS had pectoral meat that was lighter but not yellower. Since there is a link between muscle pH at 24 h and color, pH is one of the factors influencing meat color (36). Although pH values at 1-h postmortem were higher in the MAGO group, resulting in significant differences with the other water supplementation groups, for all groups, the pH values at 24-h postmortem were within the range of raw meat (5.82–5.87). These values were also similar to those reported in other studies (37, 38), which reported similar values for breast muscle. This indicates that there were no quality problems with the breast meat in any of the water additive treatment groups.

The current study found that HS increased initial pH or lightness, which contradicts previous research that found that both consistent and cyclic HS can lower pH at1 h and pH at 24 h postmortem (39, 40). These experiments may show that the duration of HS is an essential factor that can affect pH. In this study, the treatments, temperature regime, and interactions between them had an effect on the initial pH. Even if breast muscle under TN conditions at 24-h postmortem showed a higher lightness, the pH values in both raising temperatures were within the range for normal meat. The pH at 1 h postmortem was higher in the breast meat of the birds supplemented with MAGO and reared under HS due to an increased pH in the main effect of MAGO treatment and the main effect of HS. As expected, muscle pH decreased over the first 24 h postmortem. Therefore, the declining pH between the 1 and 24 h was the highest in the breast muscle of the chickens supplemented with MAGO and reared under HS due to an increased pH decline in the main effect of MAGO treatment and the main effect of HS.

The addition of betaine led to an improvement in the breast muscle, which correlated with better postmortem WHC, tenderness, and texture profile of the meat. HS reduced hot and cold carcass weight, the relative weights of breast, spleen, and heart, and meat quality. Combining MAGO with BET did not improve BET's beneficial effects on reducing the adverse effects of HS. However, when MAGO and BET were used together, the relative weights of the leg and wing showed a positive impact compared to BET alone, but the results were unsatisfactory compared to the control group.

Antioxidant activities of BET improve meat quality in birds under HS conditions (41). When subjected to HS, BET alters osmotic pressure, retains water within cells (42), and minimizes energy spent in the Na+ K+ pump, which minimizes thermogenesis from metabolic activities (43). Because a bird's body is unable to produce additional heat when heat production is reduced, deep body temperature and respiration rate decrease. We found that HS decreased initial core temperature, initial yellowness, final lightness, final yellowness, and color change—chroma, SF, springiness, and cohesiveness. HS increased dressing percentage, wing percentage, initial pH, final core temperature, initial lightness, WHC, and hardness. Because of their thermoregulatory system (covered feathers and lack of sweat glands), broilers are more susceptible to unwanted high temperatures, and global warming makes broilers especially vulnerable to HS (44). Several studies have demonstrated that chronic HS increased L^*^ values, cooking loss, drip loss, shear force, and hardness values, decreasing the springiness and pH24h and a^*^ values of the PM muscle (3, 41). The presence of water within muscle cells is crucial, as water loss can reduce the weight and thus the cost-effective value of the final meat product. It can also affect the tenderness and texture profile of the meat. The current study's findings support the findings of Fouad et al. (45) and Wang et al. (46), indicating that BET can increase breast yield, while HS decreases it because HS reduces total water content by increasing water loss, whereas BET can improve it. Heat stress likely causes membrane leakage, allowing water to escape from muscular cells. This mechanism should account for the lower total muscle water content and breast yield. Betaine, an osmolyte, prevents dehydration by preserving water in the cells and maintaining osmotic pressure (42). The present results disagree with those of Zhu et al. (47) and Abudabos et al. (19), which showed that HS increased shear force.

Texture measurements, such as SF, are made up of skeletal muscle fibers and connective tissue, and they are a method of measuring the force needed to shear connective tissue and myofibrils in a typical frame of meat. The tenderization method of meat is started by calpain-mediated fiber proteolysis of skeletal muscle, which results in smaller fragments and an increase in MFI (48). In this case, the HT group had a lower SF. However, Olson and Stromer (49) found that SF is related to other muscle indices, i.e., MFI, that were higher in the HT group. In TPA parameters, the meat is exposed to conditions similar to those in the mouth, i.e., sensory properties are measured. The HT factor worsened all parameters except chewiness. Notably, the joint use of BET and MAGO may have a variety of benefits for product quality and yield.

More research is needed to determine betaine's ability to reduce methionine addition and make betaine supplementation cost-effective, particularly when combined with different levels of methionine and choline. More research is needed to determine BETMAGO's ability to reduce BET or MAGO supplementation alone and make BETMAGO supplementation cost-effective, particularly when combined with different levels of BET and MAGO.

## Conclusion

Based on the findings, it was concluded that using betaine as an aqueous additive in broilers conferred overall benefits in terms of improvement in the carcass dressing percentage, breast weight, and meat quality in terms of water-holding capacity and tenderness without using nano-emulsified vegetable oil. However, more research is needed to explore the extended effects of nano-emulsified vegetable oil and/or betaine combinations, particularly with respect to shelf life, antioxidant activity, and nutritional value, including meat analysis, fatty acid profiles, meat composition, and microbiological analysis.

## Data availability statement

The raw data supporting the conclusions of this article will be made available by the authors, without undue reservation.

## Ethics statement

The animal study was reviewed and approved by the Ethics Committee of Scientific Research, King Saud University (KSU), Saudi Arabia (Approval No: KSU-SE-21-02).

## Author contributions

GS and EH: conceptualization. AA-O, GS, MA-G, and EH: methodology. JM: software. RA, GS, and EH: validation. RA: formal analysis. AA-O, GS, and EH: investigation. AA-O: resources and funding acquisition. HB-A: data curation. MQ, GS, and EH: writing the original draft preparation. AS: writing, reviewing, and editing and supervision. GS: visualization. GS and AA-O: project administration. All authors have read and agreed to the published version of the manuscript.
